# “Collapsing into Darkness”: An Exploratory Qualitative Thematic Analysis of the Experience of Workplace Reintegration among Nurses with Operational Stress Injuries

**DOI:** 10.3390/ijerph20176664

**Published:** 2023-08-28

**Authors:** Chelsea Jones, Brenda Juby, Shaylee Spencer, Lorraine Smith-MacDonald, Elly O’Greysik, Michelle Vincent, Colleen Mooney, Katherine S. Bright, Phillip R. Sevigny, Lisa Burback, Andrew Greenshaw, R. Nicholas Carleton, Raymond Savage, Jake Hayward, Yanbo Zhang, Bo Cao, Suzette Brémault-Phillips

**Affiliations:** 1 Heroes in Mind, Advocacy and Research Consortium, Faculty of Rehabilitation Medicine, University of Alberta, Edmonton, AB T6G 2R3, Canadasuzette2@ualberta.ca (S.B.-P.); 2 Alberta Health Services, Edmonton, AB T5J 3E4, Canada; 3 St. Stephen’s College, University of Alberta, Edmonton, AB T6G 2R3, Canada; 4 Faculty of Nursing, MacEwan University, Edmonton, AB T5J 2P2, Canada; 5 Edmonton Police Service, Edmonton, AB T5H 0H7, Canada; 6 Faculty of Nursing, University of Calgary, Calgary, AB T2N 4V8, Canada; 7 School of Nursing and Midwifery, Faculty of Health, Community and Education, Mount Royal University, Calgary, AB T3E 6K6, Canada; 8 Faculty of Education, University of Alberta, Edmonton, AB T6G 2R3, Canada; 9 Department of Psychiatry, Faculty of Medicine and Dentistry, University of Alberta, Edmonton, AB T6G 2R3, Canada; 10 Department of Psychology, University of Regina, Regina, SK S4S 0A2, Canada; 11 Canadian Institute for Public Safety Research and Treatment, Regina, SK S4S 0A2, Canada; 12 Royal Canadian Mounted Police, Edmonton, AB T5G 2T4, Canada; 13 Department of Emergency Medicine, Faculty of Medicine and Dentistry, University of Alberta, Edmonton, AB T6G 2R3, Canada; 14 Department of Occupational Therapy, Faculty of Rehabilitation Medicine, University of Alberta, Edmonton, AB T6G 2R3, Canada

**Keywords:** nursing, return to work, operational stress injury, post-traumatic stress, mental health

## Abstract

Background: Nurses are engaged in an unpredictable and dynamic work environment where they are exposed to events that may cause or contribute to physical and/or psychological injuries. Operational stress injury (OSI) may lead to an extended time away from work or nurses leaving the profession altogether. A deliberate focus on the workplace reintegration phase of the mental health recovery process may lead to the increased retention of nurses in their profession. Prior to the creation and implementation of potential solutions to address workplace reintegration, it is imperative to explore the experiences and perceptions of nurses affected by OSI. This qualitative study aims to investigate the experiences and perceptions of nurses (*N* = 7) employed within a Canadian provincial healthcare system who have attempted workplace reintegration after being off of work with an OSI. Methods: Nurses were recruited via social media, unit emails, and word of mouth. Data were collected through recorded semi-structured interviews conducted over videoconferencing. Once transcribed, the data were thematically analyzed using an inductive approach. Results: The resulting themes included (1) heroes to zeros, (2) changing the status quo, (3) connection is key, and (4) post-traumatic growth: advocacy and altruism. Study participants indicated both that nursing culture and a cumulation of events contributed to a need for a leave of absence from work and that a formalized process was desired by nurses to assist in returning to work. Conclusions: The development, implementation, and exploration of innovative policies, procedures, and initiatives to bridge the gap from clinical interventions to workplace reintegration are needed for nurses experiencing OSI. Further research is also needed regarding mental health impacts and appropriate resources to support nurses in their workplace reintegration process after experiencing psychological and/or physical injury.

## 1. Introduction

Nurses routinely work in environments that are fast-paced and unpredictable. Their dynamic work environment is constantly time-pressured, and laden with decreasing resources, staffing, shortages, ethical dilemmas, and increasingly complex caseloads [1]. Nurses regularly witness “trauma, pain, suffering and/or death” [2] in their day-to-day work, which can impact their mental health, and ability to provide safe, competent, and ethical care for their patients [3]. During the COVID-19 pandemic, nurses and healthcare professionals (HCPs) across the globe faced an influx of patients, resource limitations, system overload, policy changes, secondment, societal and political stigma, family needs, exposure to mass death and dying, as well as a personal elevated exposure risk for COVID-19 [4]. As a result, more nurses have been affected by traumatic and morally injurious events leading to increased rates of operational stress injury (OSI) and the potential need for short- and long-term leaves from work [5]. An OSI is defined as “any mental disorder or other mental health condition resulting from operational stressors experienced while serving in a professional capacity, especially in military or other public safety professions” [6]. This term encompasses a number of mental health challenges and disorders, including depression, anxiety, burnout, moral injury, and posttraumatic stress disorder (PTSD), all of which nurses are more likely to experience than other Canadians [3,6,7]. OSIs have received the most recognition in regard to uniformed professions such as the armed forces and Public Safety Personnel (PSP include, but are not limited to, border services officers, correctional workers, firefighters, paramedics, police, and search and rescue personnel) [6]. Recent discussions have also included how other professions, including nurses and HCPs, are impacted by OSIs.

Workplace reintegration after an OSI can be challenging for nurses and other HCPs. Repeated exposures to potentially psychologically traumatic situations and the physically and mentally demanding nature of a healthcare environment can contribute to career-ending challenges. An unsuccessful post-injury workplace reintegration process may impact the individual, their family, and the wider community, while further contributing to nursing shortages within healthcare systems. Further, decisions on policies, programs, and initiatives regarding workplace reintegration for nurses have not always been evidence-based, nor included perspectives of nurses with the lived experience of returning to work after injury or illness [8]. 

There is a paucity of research regarding essential post-clinical treatment when nurses are reintegrated into adverse environments and still expected to perform their occupational duties. The limited literature focusing on the return to work of nurses with mental health challenges has largely focused on military or civilian nurses experiencing substance use disorders in the United States [9,10,11]. Novel evidence-based approaches and programs can help facilitate the positive and effective reintegration of nurses into the workforce after experiencing an OSI. The lack of research specific to the workplace reintegration of nurses inhibits the development and implementation of novel initiatives. Capturing the experiences and perspectives of nurses who have engaged in workplace reintegration following injury or illness can help to address the absent research results and inform novel reintegration approaches. The current study was designed to investigate the experiences and perceptions of nurses in Alberta urban centers who have attempted workplace reintegration after being off of work with an OSI.

## 2. Materials and Methods

### 2.1. Study Design

The current qualitative thematic analysis was part of a larger mixed-methods study (QUAL quan) [11] initiative exploring workplace reintegration for HCPs and PSP.

### 2.2. Participants and Recruitment

Participants included Registered Nurses, Registered Psychiatric Nurses, Licensed Practical Nurses, or Nurse Practitioners working in urban centers in Alberta, Canada, and who were employed within a major provincial health organization. Recruitment was conducted through social media, such as Twitter and Facebook, as well as snowball and purposeful sampling. Key contacts within nursing organizations, such as peer support groups, assisted with dissemination of study flyers through their networks. Care managers of the targeted units were also asked to spread recruitment emails widely and post hardcopy flyers. Potential participants were asked to contact the research team directly and screened for inclusion before consenting to study participation. Some participants from a separate component within the larger mixed-methods study were invited by the research team to participate in the additional study. Invited participants had self-identified through an online demographic questionnaire as having required time off of work due to psychological traumatic events in the workplace and experiencing an OSI.

### 2.3. Data Collection

Data were collected between August and December 2022. This extended length of time was needed due to the staggered access to operational approval of study sites and the specificity of the participant inclusion criteria. Consent and demographic questionnaires were administered via REDCap (Research Electronic Data Capture) which is a secure, web-based software platform (Vanderbilt University, Nashville, TN, USA) [12]. Questions regarding exposure to traumatic events, mental health utilization, and work reintegration were included in the questionnaires. A semi-structured interview guide was also designed by the research team, with the goal of exploring the experience of the nurses with OSI through an inductive and deductive lens. Qualitative data collection involved 60 min individual interviews that were recorded via videoconferencing over Zoom. The research team members conducting the interviews were also registered nurses with experience in qualitative methods and mental health care, to maximize the safety and rapport of the participants. Data collection was scheduled to continue until information power was reached [13].

### 2.4. Data Analysis

The demographic and qualitative data were analyzed using Microsoft Excel software. Audio and video-recorded interviews were thematically analyzed following an iterative inductive and deductive process [14]. Initial codes were developed by identifying themes that were presented from the data. Three researchers independently conducted open coding for each interview, after which an arm’s length researcher reviewed and provided feedback on the preliminary themes and codes. The analysis of the preliminary themes was followed with discussion around any conflicting ideas among the four researchers. Once the final themes were determined, key quotes were isolated that illustrated the themes, and the final presentation of the thematic analysis was prepared.

## 3. Results

### 3.1. Demographics

Demographic details of study participants (*N* = 7) are presented in Table 1. Recruiting was open to multiple different types of nurses, but all participants were Registered Nurses. The age of the participants varied (M = 38.2, SD = 10.50), as did their years of experience in the nursing profession (M = 11.4, SD = 7.18). The average number of years the participants had worked with their current employer, the provincial health organization, was 9.3 (SD = 6.82). All participants reported exposure to psychologically traumatic events and subsequently sought mental health services. Few participants (*n* = 2) reported involvement or utilization of formal processes for workplace reintegration. At the time of data analysis, four participants had returned to work in their previous capacity, two had returned to nursing in a new role or different capacity, and one had not reintegrated back to work. It was also noted through the interviews that the time away from work varied widely amongst the participants from a period of weeks to years. The time from injury to participation in the interview also varied from a period of approximately one to seven years prior to engaging with the current study.

### 3.2. Qualitative Results

Qualitative results, while based on a small sample, exhibited dense sample specificity, and strong, focused dialogue which allowed the perceived information power to be reached with very few participants [13]. These results provide insight into the experiences of nurses returning to the workplace after a psychologically traumatic event.

**Figure 1:**
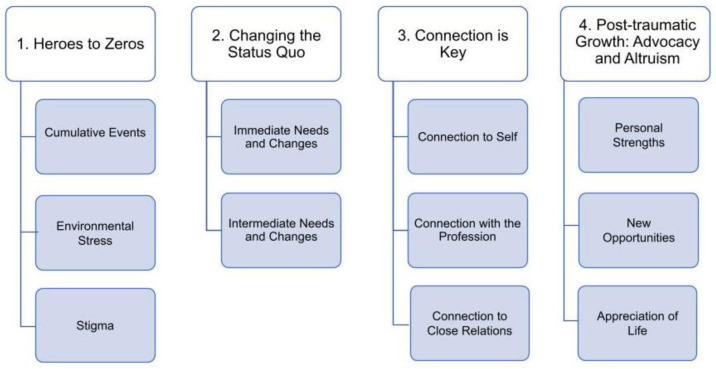
Overview of Thematic Analysis Results.

Inductive and deductive thematic analysis of the qualitative data found four themes emerge: (1), heroes to zeros, (2) changing the status quo, (3) connection is key, and (4) post-traumatic growth: advocacy and altruism (Figure 1). Threads woven within the themes included pressure from nursing culture, self-identity, and guilt. The researchers observed that the context, culture, experiences, and events that preceded time off work were important components of the participant’s stories. A description of each of these themes and sub-themes with supporting quotes follows.

#### 3.2.1. Heroes to Zeros

Nurses reported being profoundly impacted by the shift in public perception from initially being hailed as “Healthcare Heroes” at the beginning of the pandemic, to being vilified as the pandemic continued.

*“And that was when the dialogue was shifting from us being healthcare heroes to you know, that was just when the flavor really changed.”*

(P1)

The changes in public perception exacerbated the impact of exposure to potentially psychologically traumatic events, potentially morally injurious events, environmental stressors, self-stigma, and stigma from others. An under-acknowledgement of psychologically injurious events within the nursing profession by the profession, stakeholders, and the public was noted to have caused additional strain. A visual metaphor emerged through the dialogue, involving a strong and confident superhero watching pieces of their costume falling off until they are isolated and powerless.

##### Cumulative Events

An accumulation of distressing events over a period of time was reported to contribute to OSI. A single recent event was often cited as causing a “tipping point” that precipitated the need to take leave from work. Participants recognized that the dynamics of their workplace created the “perfect storm” and exposed them to both potentially psychologically traumatic events and potentially morally injurious events. Some expressed feeling a sense of helplessness and lack of control over their ability to advocate for and provide safe, competent, and ethical care to their patients, which was also a cumulative stressor.

*“… what they were doing was torturing this man. And I really felt like I was part of it. And I had, I had to take like, some time off work I just called in sick for, I don’t know, maybe maybe just like a set, but it made it so that I had like, over a week off work. Just to kind of like deal with that, because I found it really upsetting to even be like near even like the thought of going to work made me like so anxious.”*

(P3)

Subsequent care pathways utilized by the nurses did not acknowledge physical and/or mental health injuries in a holistic manner once psychological distress or an OSI was identified and reported. Participants commented that only a single psychologically traumatic event or injury was addressed at a time within the designated workers’ compensation procedures. Cumulative events were not viewed as relevant and intertwined, or compounded physical and psychological injuries were dismissed.

*“The paperwork says, well, what’s the one specific event or injury and in my mind, it’s a dozen events that are just right. And it’s not just one, it’s not just this…and it’s difficult to articulate on most forms because those forms are very regimented. For one problem, one injury, one solution done. That is not how complex psychological injuries work.”*

(P7)

Responsibility for the potentially psychologically traumatic events or potentially morally injurious events was at times (mis)placed by the organization upon the nurses who had experienced an OSI. This contributed to secondary or sanctuary/organizational trauma. Shame and guilt were experienced by some participants who felt they ought to have been able to prevent specific events from occurring.

*“I think I felt really, like I did something and it was my fault. Like, what could I have done to make this not happen? Because clearly it happens in our workplace, but you just always think, like, it’ll never happen to me kind of thing. So I’m like, What did I do? Because we were always told, like, have exits, like, keep the curtains open. And I was like, I feel like I did that. Like he was on the other side of the structure. So it was a lot of replaying, and what did I do wrong? And I think I felt kind of embarrassed about it.”*

(P2)

##### Environmental Stress

Stressful and unpredictable work environments were described by all of the participants. Staffing shortages, combined with an increasingly complex, vulnerable, and distressed caseload, were frequently cited as an overarching contributor to worsening working conditions.

*“There’s a lot of, I think, stress within the healthcare scenario. And lots of things that I find challenging when you can’t do your work properly. You’re short staffed, I feel like I’m not caring about doing the job to the best, not because of myself, but because of the scenarios around.”*

(P4)

Immense pressure to forgo time off of work including vacation, sick, and personal time was notable. Constant text messages on days off reminding them of the staffing shortages and encouraging them to pick up shifts caused participants to feel guilt when not working, even while they were at home recovering from an OSI or other injuries related to the traumatic event(s).

“But I also, there’s also that history there of being guilted to come into work, and that, you know, ignoring of concerns, our manager came to triage desk, and took off all the signs that were posted, saying like ‘this is a respectful workplace—violence and verbal abuse will not be tolerated.’ …she took down all those signs. And she said, this sends the wrong message to our patients. It’s not welcoming. And I said, ‘Well, what about when people are swearing at me, and I point to the sign saying, you can’t talk to me that way.’ And she’s like, ‘nothing bad ever happens at *site*.’”

(P7)

A healthy work/life balance was reportedly challenging to maintain in such a stressful and highly pressured environment. Consequently, participants were left feeling under-appreciated, invalidated, disrespected, and oppressed by leadership, which led to distrust of the organization as a whole. During shifts, even their basic needs, such as eating, drinking, and using the washroom, may go unmet in such a stressful work environment.

*“… in terms of trauma, and it’s like that’s what it felt like going in there. It’s like you didn’t have any idea how you were gonna make it through this shift, like, and how you were gonna be able to physically care for two people who were actively dying, you know, in trying to make sure they’re both okay. And plus, like, take care of yourself in any way like getting a break… and it’s, it is simple stuff like knowing that you can, you will be able to use the washroom when you need to use the washroom knowing like when you need water, you can get water.”*

(P3)

##### Stigma

Stigma often made it difficult for participants to acknowledge they were experiencing an OSI in the first place, or that they were continuing to feel its effects. As a result, they either denied to themselves and others that they were affected by the OSI, which often led to delays in taking time off, or premature return to work.

*“I think that really primed the pump for the PTSD. And just pretending and the denial. It’s not here. It’s not real. And kind of dissociating from what was happening. And then, the longer I stayed sick, the more angry I felt that it had happened, because I had done everything right. And I had done everything to prevent it and it still happened.”*

(P1)

By returning to work early, participants hoped for fewer questions and conversations from peers and leadership about their injury and return to work. Participants felt they had to justify their actions and decisions to all levels of the organization and all timepoints from the point of going on leave throughout their workplace reintegration. Self-stigma contributed to higher distress and emotional responses when participants had to engage in discussions or tasks regarding their workplace reintegration.

Participants discussed the guilt/pressure they felt to take medication to assist with their mental health, especially since this was not required prior to the OSI. Despite this feeling and self-stigma, some of the participants understood that medication was needed to enable them to reintegrate into the workplace. 

Stigma and discrimination in the workplace after disclosing an OSI were also experienced by some participants.

*“I’ve been in meetings where I’ve openly shared and been treated differently. I’ve been asked if I was well enough to do the work that I’m doing now by another nurse, and not in a way that was caring. Or it was in another way, that did not feel very nice.”*

(P5)

Despite internal and external mental health stigma being a reality, some participants indicated having witnessed improvements in this within the workplace in recent years. Indications of change toward healthy work environments offered some participants hope in the face of stigma.

*“It was just the thing to do. You just truck(ed) on, like, you know, like given 12 years ago, often people didn’t even want to admit if they had to take medications for depression or anything. And now that’s changing, and people are much more open about talking about it. But 12 years ago, there was still a fair bit of stigma regarding it.”*

(P4)

#### 3.2.2. Changing the Status Quo

Several immediate and intermediate needs and desired changes regarding responses to potentially psychologically traumatic events and the workplace reintegration process were identified by the participants. This included recommended changes to existing policies, practices, and procedures from a point of stress through to workplace reintegration. Education at all levels of the organization related to mental health, trauma-sensitive practice, coping strategies, and profession-specific mental health resources was specifically identified as being needed both immediately after a traumatic event, and intermediately for the affected staff, their peers, family units, and leadership. Increased education was cited as potentially leading to enhanced validation of nurses ‘experiences, understanding of their injury, and hope for successful work re-entry.

*“Education or mental health resiliency, and all these things that we’re lacking, are more important than taking care of our patients. If we’re not healthy, our patients aren’t going to be well either.”*

(P7)

Compassionate, empathetic, respectful, engaged, and supportive leadership, as well as non-judgemental colleagues, was also identified as integral to recovery and reintegration both immediately and intermediately.

##### Immediate Needs and Changes

A standardized process and protocol for acknowledging and responding to potentially psychologically traumatic events or potentially morally injurious events was desired by participants as a means of addressing gaps in the current processes, structures, and supports.

*“I think there is no clear process for acknowledging when somebody has had a psychological injury. Yeah, so not the same way as if I’ve had a back injury. There’s a very clear return-to-work process (for physical injury).”*

(P1)

Acknowledgement by the healthcare organization of the risk to nurses of experiencing psychologically traumatic and morally injurious events was strongly desired by the participants. Debriefing immediately after the injurious event was proposed to facilitate the processing of the event and learning about both signs of mental health challenges and nursing-specific mental health resources.

*“I don’t think anybody ever talked about anything. Except my co-workers were very upset because, they were like, this could happen to anybody in our department. And I think they wanted to hear something from managers, they wanted to have some sort of debriefing or some sort of ability to talk about kind of what happened.”*

(P2)

##### Intermediate Needs and Changes

A formalized individually tailored return-to-work process was desired by the participants. Current systems for navigating return to work after an OSI required participants to be action-oriented at a vulnerable time, leaving them to navigate complex systems with limited, if any, assistance.

*“I wouldn’t kind of just work myself so hard. And that’s what I felt that I needed to do last time. But I’m sitting here as you ask these questions, I’m like ‘Why? Why did I have no idea but there wasn’t anyone directing the kind of time off or the comeback other than me so I was the one making those decisions?”*

(P6)

An interdisciplinary workplace reintegration program that was individualized, paced, and led by the reintegrating individual was envisioned by participants. Personalized programming was seen as essential to facilitating safe return to work. Reintegration at the right time was also seen as critical to minimizing retraumatization and reducing delay to overall recovery and re-entry into the workplace.

*“But I think had I been offered, like, some kind of a slower, like, reintroduction to work, which I know would have been really hard to support, especially like last summer. But that would have been helpful, I think, because it was… it was really tough to go back to, like, full time caring for it.”*

(P3)

Access to financial support to compensate for lost wages and sick time benefits would also enable those with OSIs the time they need to recover and prevent premature workplace reintegration. 

The development of policies and procedures regarding workplace reintegration was desired by participants. They felt this may reduce duplication of services and inadequate communication between the services, organization’s clinicians, and OSI-affected nurses. Presumptive legislation for nurses affected by OSI was also strongly advocated for by the participants so as to reduce the distress caused by the need to “prove” that an OSI was, in fact, a result of their work.

“… * the province* not having presumptive PTSD legislation I had to go through an extensive process. I had to go through a comprehensive psychological assessment. But of course, it was five months after the fact.”

(P7)

#### 3.2.3. Connection Is Key

Connection with self, the profession, peers, chosen family, and community were identified by participants as all being necessary for recovery from the OSI and subsequent workplace reintegration. A team-driven and collaborative workplace nurtures healthy working relationships, but this was not what many of the participants experienced. Many participants lamented the lack of healthy workplace relationships and commented that having these relationships was and would be central to workplace reintegration and recovery from OSI.

##### Connection to Self

A loss of self-identity and purpose contributed to participants becoming overly entrenched in their work roles once they were injured and could no longer engage in professional activities.

*“When you’re in the darkest moments of this…there’s an identity crisis, there’s like, “am I ever going to nurse again?” and “what the hell is happening to me”, like you just, it feels like, just a cloud, you’ve lost a piece of yourself, you don’t know who you are anymore.”*

(P5)

*“My guilt for being home was crippling. And I don’t say that lightly. But it was because here I am. I’m like ‘here, I’m an emergency nurse. This is my passion, my profession. This is what I’ve been trained for. And here I am at home crying when my coworkers are suffering’.”*

(P7)

After recovery, the participants could acknowledge that stepping away from work allowed them to gain insight into themselves, their personal identities, and their work environments. A central realization was that they had a right and responsibility to care for themselves, and there was more to who they were than their role and profession as a nurse.

*“And I guess taking that time off. And like, losing touch with that, I guess, like not not being able to consider that kind of like my primary, a primary part of my identity or having to think like, will I be able to stay in ICU? Like, do I need to take a different type of job and like, this is what I love. I guess I kind of realized that maybe I need to, like take a step back and kind of take, you know, I can care about what I do. But it’s also… it is just a job. And I need to, like, prioritize myself over any of this.”*

(P3)

##### Connection with the Profession

Connection within the profession was cited by participants as being important to facilitate a compassionate, respectful, validating culture, and environment. A lack of connection between peers, leadership, and workers’ compensation organizations resulted in them experiencing additional distress, distrust, and isolation. Participants felt that dismantling silos between the union, leadership, workers’ compensation organization, and staff would be beneficial to addressing OSI-related concerns.

*“And no one even, from management or anything, even like checked on me, which I really felt, like, annoyed and upset about at the time. But now I’m kind of like, ‘I don’t know’. If they…don’t know what they’re kind of, like, allowed to do in terms of like, not overstepping boundaries, but it felt kind of like, insensitive, like, no one really said, like, “Oh, how are you feeling? Are you okay?” Or whatever. It’s just like…I didn’t even leave…my work friends, the people I work with were really good…they were really supportive.”*

(P6)

*“My manager, who had just come in, I had never met her before you emailed me and said, I know what happened to you. I’m really sorry, I’m here. These are some mental health resources. I’m a big proponent of it. She was great. Rather than the previous cadre who had just kind of slid the brochure across the table, you know.”*

(P1)

Formal or informal peer support with nurses who understand the turbulent workplace demands were also cited by participants as helpful.

*“And she (colleague) checks on me like she, we kind of check on each other. She’s someone that like, seems to cope really well with all this stuff. But she’s someone I can definitely, like, count on. And then I know that like, if I were to ever just tell my friends like I’m having a really bad day or whatever…they would be there for me.”*

(P3)

##### Connection to Family and Friends

Positive support outside the workplace was emphasized by the participants as important to recovery and workplace reintegration; nevertheless, some participants reported feeling responsible for keeping family and friends unburdened by their mental health challenges. Friends and family may have been the first to identify behavioral changes within the participants, which helped them identify their own mental health challenges and seek help. In some cases, participants reported their closest relations were unavailable or incapable of providing support, which created additional barriers to recovery and reintegration.

“I told my parents, my parents don’t live here. And from *province* my dad, and my mom. And my brother is my only family who lives here. And he said, “You know, I don’t know if this job is the best for you.” I’ve always had lots of family and friends around me, so I’m blessed in that way. And, they also noticed that I was isolating. So my husband, you know, we were just keeping it together…I said to him, finally, I’m like, you got to go and talk to someone because, like, I know, this was hard on you.”

(P5)

*“[M]y partner unfortunately wasn’t…I don’t think he really even had the capacity to be and then like, also, you know, living with someone that’s depressed and anxious and stuff, and not knowing how to deal with that is probably not easy either. So yeah, that, that I ended up ending that relationship.”*

(P3)

Close relations further assisted participants with navigating and accessing assistance before or after leaving work.

*“I think having somebody there to be able to talk to you, that’s a safe person. Yeah. And I would say, like, really reach out to your support system, because I don’t know what I would do without them. Yeah. I genuinely, like, that sounds very scary, but I don’t think I would still be here without them. I think I would have, like, I think I really would have collapsed in the darkness.”*

(P2)

#### 3.2.4. Post-Traumatic Growth: Advocacy and Altruism

Post-traumatic growth was a common theme identified by participants. Despite the challenges nurses experienced during their OSI recovery and subsequent workplace reintegration efforts, it helped them to recognize their personal strengths and resilience, engage in new opportunities, and find a new appreciation in their profession and personal lives. Many participants wanted to altruistically assist other nurses in their recovery from OSI, through formal and informal peer support, and advocate for change within systems and organizations.

##### Personal Strengths

Coping and emotional regulation skills were strengths that participants identified as having increased during their recovery. The importance of self-advocacy skills and stronger boundary settings was also emphasized, with the goal of protecting and enhancing their mental health and wellness at work and home.

*“Some of the differences is that I will realize when I’m getting low, that I will necessarily take a mental health day versus…not. Not having a clue that there’s days when I just know, mentally I need to do that. And I would say there was definitely days when I was redeployed to ICU that I did take more mental health days last fall… I just, I knew I’m like, that’s it. I can’t come back to this scenario tonight. I have realized that sometimes there’s things that I’m just like, need some time out?”*

(P4)

Participants acknowledged and accepted the importance of addressing mental health challenges by developing self-awareness, education, and engagement with mental health treatments. Participants also described enhanced compassion and empathy for others experiencing mental health challenges as having being bolstered during their recovery.

“So working on that with people and just kind of being an advocate for workplace safety. And helping some of my co-workers and another co-worker got punched in the face by a kid. And just like helping with like, K, you need to do this*workers compensation organization*, you need to go get checked out by a doctor and just kind of being somebody there for support and the department.”

(P2)

*“I have a lot more empathy. For everybody with every mental health concern, I always did. But man…”*

(P1)

##### New Opportunities

Some participant experiences with OSI and workplace reintegration proffered new opportunities for growth; for example, gaining new knowledge, pursuing education, moving into alternate nursing positions, finding new leisure activities, making new connections, and engaging in other paid, volunteer, or informal roles that assist others in the community. Participants identified a sense of peacefulness with regard to finding their voices and cumulatively advocating for support for one another.

*“I don’t have to continue in emergency if I don’t want to. I don’t need to be a trauma nurse. I really wanted to be a trauma nurse… But now I’m kind of like, I don’t want that stress in my life. And I will be okay. Without that I don’t need that; I will kind of do the work that makes me happy.”*

(P2)

*“I am now driven and very passionate for supporting mental health for nurses. And I have a lot of experience and knowledge that I want to share with other nurses because there is a substantial gap of knowledge within our profession, and that needs to be addressed, and it’s not in a negative way.”*

(P7)

##### Appreciation of Life

Participants reported shifts in their personal priorities and enhanced self-determination after being away from work and experiencing an OSI. Participants also reported renewed appreciation of life and time spent with family and friends. Engaging in purposeful activities became clearer and prioritized among participants as a result of their recovery journey.

“… [I]t almost kind of feels like I don’t owe *healthcare organization* my life anymore. Yeah, I feel like I’ve kind of gotten into being a nurse is not my whole life. There are other things to life that matter. So now I will take my personal days if I need to.”

(P2)

Having a sense of purpose beyond their nursing career resulted in participants feeling less guilt when taking time off of work.

*“I can happily say that I am very grateful for the experiences that I’ve had, because I have this new knowledge I would have never had. And that’s, you know, someone who’s talking that’s done a lot, a lot of work and healing. And, you know, so there’s a lot of gratitude there.”*

(P5)

## 4. Discussion

The current study was designed to explore the experiences and perceptions of nurses (*N* = 7) employed within a Canadian provincial healthcare system who have attempted workplace reintegration after being off of work with an OSI. The current study appears to be the first to specifically address the topic of workplace reintegration among nurses with OSI. Participant engagement helped to contextualize the current nursing context, factors that contribute to OSI, and facilitators, barriers, and changes needed for workplace reintegration among Canadian nurses. 

The first theme identified factors that contributed to OSI and workplace reintegration, which included potentially psychologically traumatic events, potentially morally injurious events, environmental stressors, or stigma. Within the healthcare environment, staffing shortages in the midst of higher and more complex caseloads appeared to underlie many of the professional and personal challenges experienced by nurses. Perceived and experienced internal and external stigma may also cause nurses to ignore mental health symptoms or equate seeking help with personal weakness [15]. The stigma may also be perpetuated by “long standing occupational cultures and organization leadership that continue to reinforce the perception that mental health conditions such as PTSD are a sign of weakness” [2]. Nurses often reported feeling immense pressure to be strong and act like “heroes”. Expectations from the public, organizations, and other nurses reinforced the perceived imperative to be strong and stoic in times of hardship. 

The second theme identified barriers to recovery from OSI and workplace reintegration, and desired changes to these processes. The nurses often felt they lacked support after psychologically injurious events, and did not receive timely psychoeducation and resources that may have assisted them with their subsequent mental health challenges. Suggestions to improve upon these processes included changes in policies, practices, and procedures immediately following a traumatic event(s), starting with an acknowledgement that a potentially psychologically injurious event has taken place. Consistent policies and procedures for providing support to staff during and after these events were desired by participants, together with compassionate leadership and non-judgement from peers. Some participants felt abandoned by their health organization to navigate their workplace reintegration independently without standardized policies, programs, support, or resources, which may have contributed to poorer return-to-work outcomes. Only two participants had engaged in a formalized workplace reintegration effort. The need for education on mental health through a trauma-sensitive lens at all levels of the organization as well as for those nurses who experience OSI was noted as a prominent need, which is consistent with recommendations from the literature [8,16]. Critical incident debriefing following a potentially psychologically traumatic event was advocated for by some of the participants; however, the available effectiveness evidence for this practice remains insufficient based on research regarding PSP [17,18]. Participants also noted that addressing intermediate needs may facilitate effective work reintegration processes. A tailored approach to return to work, support post-clinical intervention, respect and autonomy from peers and leadership, and adequate time and financial support to remain off work as needed were highlighted as essential. This is consistent with recommendations in previous publications specific to nurse’s return to work after experiencing a substance use disorder [8] and the literature regarding workplace reintegration of PSP [19,20]. 

The third theme identified and emphasized the importance of finding and leveraging connections within self, the profession, family, and friends during their recovery journey and workplace reintegration. Social support has been shown in the literature to be an important aspect of resilience and recovery after psychologically injurious events or psychological distress via a number of psychological and behavioral mechanisms. These include motivation to adopt healthy lifestyle behaviors and reduce risky behaviors, feelings of being understood, appraisal of potentially stressful events as being less threatening, enhanced sense of control or mastery, increased self-esteem, use of active coping strategies, and impact of social influence and social comparison [21]. Participants redefined their identities and found a deeper understanding of their purpose and how that related to their nursing role. Connecting with peers provided a sense of strength and support; connections in the workplace may contribute to a more productive, collaborative, and efficient workplace which allows nurses to provide the best care possible to their patients. Participants emphasized connections with family and friends as facilitating recovery and return to work, which was consistent with the extant literature [10,16,18]. 

For the fourth theme, post-traumatic growth was identified in three of the five domains of the formal definition of this construct [22]. These included (1) recognition of personal strengths, (2) new opportunities, and (3) appreciation of life. Participants reported coming to a point of acceptance of the psychologically injurious events in which they were engaged, their OSI, and the recovery journey that transpired as a result. The participants were empowered to make a number of life changes as a result of their post-traumatic growth which included setting better boundaries, seeing the importance of other aspects of life, and engaging in activities that give back to the community. Altruism was an important factor, and participants felt motivated to participate in the current study to advocate for other HCPs and participate in advocacy initiatives for other nurses with OSI. 

### 4.1. Recommendations

The study results informed several recommendations regarding the workplace reintegration of nurses who have sustained an OSI. The recommendations were related to addressing OSIs, policy, initiatives and implementation related to workplace reintegration, and future research.

#### 4.1.1. Addressing OSI

The nursing profession, educators, leadership, healthcare organizations, government agencies, workers’ compensation organizations, and the public at large need to acknowledge that nurses experience potentially psychologically traumatic events. Without such acknowledgement, nurses will continue to have invalidating experiences that cause additional, unwarranted distress and bureaucratic burden after sustaining an OSI. Unions, healthcare organizations, mental health advocates, and governments could educate stakeholders and the public and advocate for global awareness and acceptance that nurses experience potentially psychologically traumatic events. The proposed psychoeducation may help reduce the secondary and sanctuary/organizational traumas experienced by injured nurses. The reductions may also help to reduce injury severity, shorten workplace reintegration processes, and increase retention.

Additional education related to profession-specific mental health, including a focus on trauma and moral injury, wellness, and coping strategies for all levels of the healthcare organization, is recommended. This would assist nurses and their networks to increase mental health literacy and reduce stigma [8,10,23,24]. A trauma-sensitive approach is recommended for developing and delivering educational programs and training [2]. The delivery of mental health and wellness education should be provided regularly and at different points of the nursing career trajectory. It is recommended that further resources from healthcare organizations and government be allocated for the development of such education. Many such resources may include online and in-person resources and methods of delivery [3]. Finally, the increased engagement of leadership and organizational support for nurses from the point of OSI recognition through recovery and reintegration is recommended [8,16,25]. 

#### 4.1.2. Workplace Reintegration: Policy, Initiatives, and Implementation

The revision and creation of evidence-guided policies, programs, practices and initiatives are needed to further support nurses in workplace reintegration after an OSI, and to bridge the gap from clinical interventions for OSI to a full workplace reintegration. Setting the stage through education on the challenges and importance of workplace reintegration after clinical interventions may be an initial step in this process. Such education would be appropriately aimed at stakeholders, such as nurses, nursing students, nurse educators, leadership, administrators and policymakers, workers’ compensation organizations, varying levels of government, unions, and HCPs engaged in the care and rehabilitation of nurses. 

Novel policies, programs, practices, and initiatives will require context-specific development, design, implementation, fidelity, and evaluation efforts to ensure effectiveness. Initiatives must involve a formalized, well-communicated process that can be individually tailored to be person and profession-specific. Individuals must be provided with the space and time needed to recover and engage with a clinical intervention addressing the OSI without pressure to return to work too early. Allowing the individuals to have control and choice is imperative for a successful workplace reintegration, enabling the nurses to feel empowered. The incorporation of formal peer support into such efforts is also recommended based on the results of this and other studies [8,10,16,17,19,20,23,24,25].

Workplace reintegration and return-to-duty initiatives by PSP and military organizations may provide a template that could be contextualized and adapted to a healthcare environment [9]. Healthcare organizations would benefit from working collaboratively with external researchers to further inform approaches to workplace reintegration. Active, community-engaged processes that capture and validate the experiences of nurses are also important in the design, implementation, and evaluation of incoming initiatives [26,27]. Engaging and collaborating with the stakeholders at micro, meso, and macro levels, through clear communication policies, plans, and practices, will also facilitate stronger initiatives and implementation of initiatives [20]. Finally, the use of the effective implementation of scientific approaches could best facilitate changes to current practices [28].

Presumptive legislation adoption and implementation by governments and healthcare systems is strongly recommended. Presumptive legislation “accepts disease or disorder claims from a worker, such as PSP, without the worker having to prove that the physical or psychological disease or disorder resulted from the job” [29]. This legislation would enable nurses and other HCPs with OSI to attain clinical and financial support in a more immediate and streamlined fashion, thereby reducing the administrative and advocacy burden they experience at a time of increased distress and vulnerability.

#### 4.1.3. Future Research

Research is needed in multiple areas related to workplace reintegration and OSI among nurses and HCPs. First, more research and advocacy are recommended regarding the healthcare settings as the effects of chronically stressful environments are modifiable risk factors for OSI in HCPs and nurses. Secondly, listening to and researching the perspectives and experiences of nurses and HCPs who have experienced OSI and engaged in workplace reintegration are critical to developing interventions that are effective, co-designed, and population-specific. The existing literature also advocates for research to consider gender more carefully. This is particularly important given that nursing is a woman-dominated profession resulting in research that is biased by the homogeneous nature of participants [8,30]. Gender, education, and other demographic factors may affect cultural beliefs and stigma regarding OSI. Further, research regarding OSI and workplace reintegration that considers other HCPs that are exposed to potentially traumatic and morally injurious events, such as respiratory therapists, doctors, social workers, rehabilitation professionals, mental health practitioners, etc., is also warranted. 

As mentioned, workplace reintegration initiatives will need to be visioned, developed, implemented, and scaled to address this issue. Once initiatives are started, high-quality studies need to explore the effectiveness, efficacy, safety, and fidelity of novel policies, procedures, or programs [20]. When researching policies, practices, and initiatives, workplace reintegration research should incorporate constructs and conditions which may correlate with success in sustained return to work, such as measures of absenteeism, presenteeism, organizational injustice, work function and performance, perceived stigma, as well as mental health knowledge and attitudes [17,20]. Studies that address the cost-effectiveness of policies, procedures, initiatives, and programs for workplace reintegration will also be needed to determine if these are effective in reducing disability costs at the individual, organizational, and community levels. 

### 4.2. Strengths and Limitations of Study

The current study acknowledged, listened to, and amplified the voices of nurses with lived experience of OSI and workplace reintegration. The results included identifying barriers, facilitators, and recommendations for return to work to be identified. The current study also captured a glimpse into the COVID-19 pandemic experiences of nurses, which will be retrospectively viewed as an important point in the history of healthcare systems as a whole. 

There were several limitations to the current study that can inform future research. Recruitment for the current study was lower than expected, likely due to the sensitive nature of the topic and the demands currently placed on healthcare professionals and systems as a result of the COVID-19 pandemic. Interviews were commonly rescheduled or cancelled completely as potential participants attempted to accommodate shift changes, overtime, and other demands. The scheduling challenges resulted in a smaller-than-expected sample size, limiting generalizability. Additionally, gender bias is notable in this study. Although efforts to recruit all genders were made, nursing remains a profession of mostly women and this was reflected in the data [30]. Finally, participants self-identified as experiencing an OSI and this was not further verified with medical records.

## 5. Conclusions

Nurses and other HCPs are frequently exposed to potentially psychologically traumatic events and potentially morally injurious events. Profession-specific and tailored policies, practices, initiatives, and support are needed to assist HCPs in OSI recovery and subsequent workplace reintegration. HCPs should not be compelled to return to work in a nondescript capacity, but should be enabled to reintegrate with purpose into their desired roles and capacities and be retained long term. Listening to and amplifying the voices of nurses with lived experience of OSI and workplace reintegration allowed for barriers, facilitators, and recommendations for return to work to be identified. The current results can assist in setting the stage for subsequent work on the development and evaluation of novel approaches to best serve nurses reintegrating into the workplace. Development, implementation, research and exploration of innovative policies, procedures, and initiatives can help bridge the gap from clinical interventions to workplace reintegration for nurses experiencing OSI.

## Figures and Tables

**Table 1:** Results of the Participant Questionnaire (*N* = 7).

Participant Demographics		Frequency (*n*/%)
Sex	Female	7/100
	Male	0/0
	Intersex	0/0
	Prefer not to say	0/0
Gender	Woman or feminine	7/100
	Man or masculine	0/0
	Transgender man, male, or masculine	0/0
	Transgender woman, female, or feminine	0/0
	Gender nonconforming, genderqueer, or gender questioning	0/0
	Two-spirit	0/0
	Prefer not to specify	0/0
Age (years)	18–24	1/14
	25–34	1/14
	35–44	1/14
	45–54	2/29
	55–64	0/0
	65+	0/0
	Prefer not to say	2/29
Ethnicity	White	6/86
	South Asian (e.g., East Indian, Pakistani, Sri Lankan, etc.)	1/14
	Chinese	0/0
	Black	0/0
	Filipino	0/0
	Latin American	0/0
	Arab	0/0
	Southeast Asian (e.g., Vietnamese, Cambodian, Laotian, Thai, etc.)	1/14
	West Asian (e.g., Iranian, Afghan, etc.)	0/0
	Korean	0/0
	Japanese	0/0
	Indigenous, Metis, Inuit	0/0
	Other/Unknown	0/0
	Prefer not to say	1/14
Highest Level of Education	High School Diploma	0/0
	Vocational or technical college	0/0
	College Diploma	0/0
	Some undergraduate	0/0
	Undergraduate degree	5/71
	Graduate degree	2/29
Professional Role	Registered Nurse	7/100
	Registered Psychiatric Nurse	0/0
	Licensed Practical Nurse	0/0
	Nurse Practitioner	0/0
Years in Profession	0–4	2/29
	5–9	1/14
	10–14	2/29
	15–19	1/14
	20–24	1/14
	25–29	0/0
	30+	0/0
Years with Provincial Health Organization	0–4	2/29
	5–9	1/14
	10–14	3/43
	15–19	0/0
	20–24	1/14
	25–29	0/0
	30+	0/0
Psychological Exposure to Trauma	Yes	7/100
	No	0/0
	No Answer/Prefer not to say	0/0
Psychological Distress as a Result of Trauma Exposure in the Workplace	Yes	7/100
	No	0/0
	No Answer/Prefer not to say	0/0
Sought Mental Health Care as a Result of Trauma Exposure in the Workplace	Yes	7/100
	No	0/0
	No Answer/Prefer not to say	0/0
Time off Work Due to Exposure to Psychological Trauma in the Workplace	Yes	6/86
	No	0/0
	No Answer/Prefer not to say	1/14
Participation in a Workplace Reintegration or Return-to-Work Process	Yes	2/29
	No	5/86
	No Answer/Prefer not to say	0/0
Return to Work Status	Full-time, previous role	4/57
	Part-time, restricted duties, and/or different role	2/29
	Have not returned to workplace	1/14

## Data Availability

Data may be available upon request directly from the authors. Availability will be decided upon on a case-by-case basis with the author’s discretion to maximize the anonymity and confidentiality of the participants in this study.
